# Tubulocystic Renal Cell Carcinoma of the Native Kidney in a Renal Transplant Recipient: A Rare Case Report

**DOI:** 10.1155/2020/7145652

**Published:** 2020-10-14

**Authors:** Brianna Ruch, Ashley J. Limkemann, Paulo Garcia, Cameron Benedict, Gaurav Gupta, Marlon Levy, Amit Sharma

**Affiliations:** ^1^Hume-Lee Transplant Center, Virginia Commonwealth University, Richmond, VA, USA; ^2^Department of Pathology, Virginia Commonwealth University, Richmond, VA, USA; ^3^School of Medicine, Virginia Commonwealth University, Richmond, VA 23298, USA

## Abstract

Tubulocystic renal cell carcinoma (TCC) is a rare and newly recognized variant of renal cell carcinoma, which may mimic benign cystic disease of the kidney. To our knowledge, we present the first reported case of a patient who, despite standard preoperative workup, developed TCC of his native kidney soon after receiving kidney transplantation. He was appropriately treated with native nephrectomy and has had no signs of reoccurrence 7 years postoperatively. Given the significant risk of malignancy in renal transplant patients, this case emphasizes the need for close monitoring of native cystic disease before and after transplantation, with low threshold to proceed with surgical intervention.

## 1. Introduction

Tubulocystic renal cell carcinoma (TCC) of the kidney is a rare variant of renal cell carcinoma (RCC), and it was not recognized as a distinct entity by the World Health Organization until 2016 [1]. Literature review only yields approximately 100 reported cases, the majority of which focuses on histologic features and differentiation. There are no reported cases of TCC in transplant recipients, despite the high risk of renal cancer in this population [2]. We report a rare case of tubulocystic renal cell carcinoma occurring in the native left kidney of a renal transplant recipient.

## 2. Case Presentation

**Figure 1:**
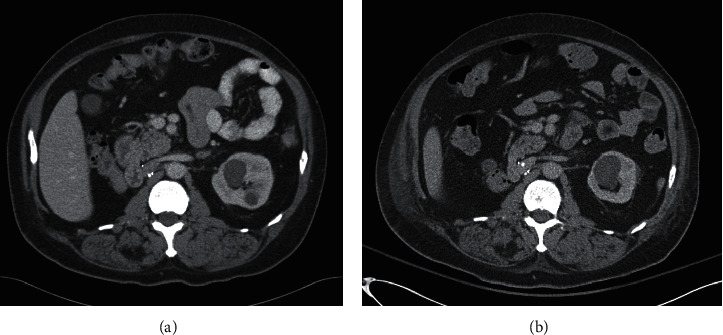
Contrast-enhanced CT scan of the abdomen (a) two years before transplant and (b) five months after transplant. (a) Left kidney showing a 3.1 × 2.6 cm, nonenhancing, cystic lesion at the upper pole. (b) Enlarged and minimally enhancing 3.3 × 3.8 cm mass in the upper pole of the left kidney suspicious for renal cell cancer (white arrow).

A 65-year-old African American male underwent a native right nephrectomy for RCC 25 years ago. Five years later, he began hemodialysis for end-stage renal disease (ESRD) secondary to hypertension and insulin-dependent diabetes. As part of a workup for renal transplant consideration, he underwent contrast-enhanced computed tomography (CT) of the abdomen in 2010 that demonstrated multiple low-attenuation cysts, too small to characterize, and a 3.1 cm × 2.6 cm nonenhancing lesion in the left renal upper pole consistent with a Bosniak class II renal cyst. Based on these findings, no further follow-up was recommended (Figure 1(a)). Subsequently, he underwent a living-related kidney transplant in 2012 with an immunosuppression regimen consisting of rabbit antithymocyte globulin (thymoglobulin; Genzyme Corp., Cambridge, MA) for induction (day 0 to day 3 at 1.5 mg/kg) and maintenance therapy of tacrolimus, mycophenolate mofetil, and a tapering dose of prednisone.

**Figure 2:**
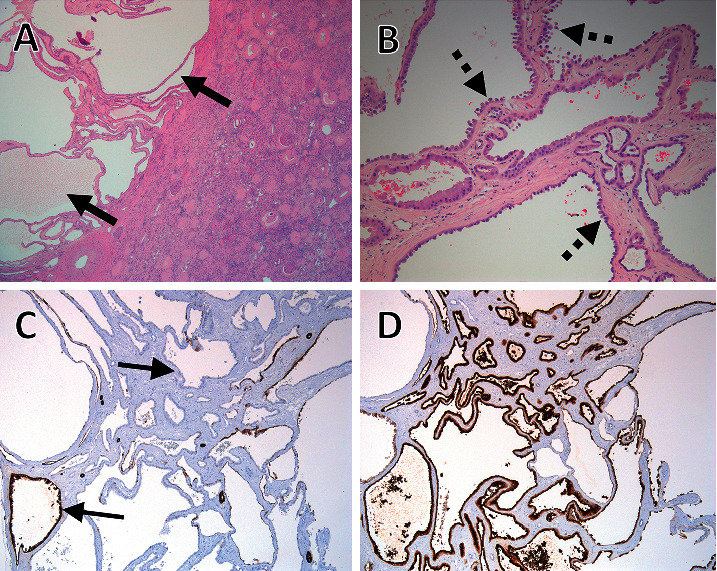
(a) Well-demarcated lesion of small- to intermediate-sized tubules admixed with cystically dilated tubules (solid thick arrows). (b) Tubules and cysts comprising a single epithelial layer of flat to hobnail-shaped cells with abundant, pink cytoplasm and large nuclei with prominent nucleoli (dashed thick arrows). The stroma was fibrotic and paucicellular, while mitotic activity was low to absent. No papillary architecture or epithelial cell clearing was observed. (c) Immunohistochemical stain for CK7 showed patchy reactivity (thin solid arrows) in the lesional areas. (d) CD10 showed strong reactivity.

The patient presented to the clinic five months after his transplant with complaints of hematuria and left leg swelling. Abdominal CT scan revealed an enlarging, minimally enhancing hypodense 3.3 × 3.8 cm cystic mass, Bosniak class IV, in the upper pole of the native left kidney that was suspicious for RCC (Figure 1(b)). Venous duplex of the left leg demonstrated deep venous thrombosis extending from the popliteal to the left common femoral vein. Patient underwent thrombolysis, placement of the inferior vena cava filter, and systemic anticoagulation followed by an uneventful laparoscopic left radical nephrectomy. Macroscopic evaluation of the specimen showed a 2.3 × 2.1 × 2.0 cm well encapsulated, polycystic mass in the superior pole of the left kidney abutting the pelvis but not invading the renal parenchyma. Immunohistochemical analysis of the neoplastic cyst-lining cells was positive for racemase, CD10, vimentin, and focal positive staining with CK7, as well as negative staining with EMA (Figure 2). The patient was diagnosed with tubulocystic renal cell carcinoma of the kidney, Fuhrman nuclear grade 3, confined to the kidney.

Postoperatively, our patient was followed per the 2018 National Comprehensive Cancer Network (NCCN) guidelines with abdominal imaging at one year and chest imaging annually over three years [3]. He was considered tumor-free based on his last abdominal magnetic resonance imaging in 2014, which showed no recurrent or residual disease. He continued to do well, about seven years after his renal transplant and left native nephrectomy, with a serum creatinine of 1.59 mg/dL and eGFR 49 mL/min/1.73 m^2^. His current immunosuppression consists of tacrolimus 2 mg twice a day, mycophenolate mofetil 750 mg twice a day, and prednisone 10 mg daily.

## 3. Discussion

Cystic lesions of the kidneys may present a diagnostic dilemma in ESRD patients, especially if they are being considered for renal transplantation. Tubulocystic renal cell carcinoma (TCC) of the kidney is a rare, recently recognized entity of renal cell carcinoma (Table 1) [1, 4–7]. To our knowledge, TCC occurring within the native kidney of a renal transplant recipient has not been previously reported.

Tubulocystic renal cell carcinoma is often diagnosed incidentally. However, some patients may present with hematuria, leg swelling, or abdominal pain [8]. It occurs most frequently in the fifth or sixth decades with a strong male predominance (male : female ratio: 7 : 1), and approximately 60% occur in the left kidney [2]. The differential diagnosis of TCC includes other tumors with cystic appearance on imaging, such as multilocular cystic RCC and adult cystic nephroma [7]. It is not uncommon to have TCC described on imaging as cysts with unusual or atypical features [9] and can vary in appearance from Bosniak class II to IV [7]. Macroscopically, TCCs are well-circumscribed lesions comprising small cysts with multiple thin septa that give them a “bubble wrap” appearance. Although relatively indolent, TCC can behave aggressively, and it should be treated with partial or total nephrectomy [10]. Recurrence and/or metastasis occur in less than 10% of cases.

Renal transplant recipients have an increased risk (0.5–1.0%) of developing renal cancer when compared to those with ESRD (0.3%) and the general population (0.005%) [11–14]. The highest incidence of renal cancers occurs in the native kidney but may also occur in the renal allograft or even be transmitted from the donor [15]. This makes renal cancer the third most common solid tumor in transplant recipients and accounts for 10% of all cancer-related deaths following renal transplantation [16]. Risk factors for RCC in renal transplant recipients include the length of time on dialysis, the presence of renal cysts, male sex, and African descent [17, 18].

Most patients who have been on dialysis more than 3 years will have acquired renal cysts, of which 2% will develop RCC (11). These renal cysts may be categorized based on their radiologic characteristics (septa, cyst content, and enhancement) into Bosniak I, II, IIF, III, and IV classifications [19, 20]. The Bosniak classifications stratify malignancy risk, with a recent 2016 review finding malignancy occurring in 9% of type II cysts, 18% of type IIF, 51% of type III, and 86% of type IV [21]. Standard practice recommends no further intervention for Bosniak I and II, interval imaging follow-up for Bosniak IIF, and surgical intervention for Bosniak III and IV cysts [20].

Despite the increased frequency of renal cysts and RCC in patients with ESRD, there are no widely accepted screening protocols or lower interventional thresholds either before or following transplantation [11, 22, 23]. The lack of screening can be attributed to the relatively high mortality associated with ESRD and the fact that screening has not been shown to significantly increase life expectancy except when performed in younger patients. Currently, many dialysis centers will only perform routine renal screening on patients who are on the transplant waitlist [22]. For ESRD patients on the renal transplant waitlist (or undergoing evaluation) that have suspicious or complex cystic lesions, we suggest a nephrectomy in (a) young patients with a history of long-standing dialysis, (b) prior history of renal cell carcinoma in the contralateral kidney, and (c) those who may be expected to follow-up with their local nephrologists for long-term posttransplant care.

We conclude that tubulocystic renal cell carcinoma is a distinct entity of RCC. This rare case report highlights the elevated risk of developing renal cancer in transplant recipients, especially those with known renal cystic disease prior to transplantation. Therefore, such high-risk recipients should undergo either close postoperative monitoring, with annual surveillance ultrasound [16], or undergo a native nephrectomy prior to transplantation.

## Figures and Tables

**Table 1:** Common subtypes of renal cell carcinoma [3–5].

Renal cell carcinoma	Associations	Imaging	Morphologic characteristics	Behavior	Incidence
Clear cell	Patients >50 years Von Hippel–Lindau tuberous sclerosis	Hypervascular Heterogeneous (hemorrhage, necrosis, or cysts) Originates from the renal cortex	Cells with clear cytoplasm, prominent nucleoli, and extensive intricate branching vasculature, arranged in a nested architectural pattern.	Aggressive	75%

Papillary cell	Patients >50 years Hereditary papillary RCC End-stage renal	Hypovascular Homogeneous More common to be bilateral or multifocal	Neoplastic cells arranged in discrete papillary fronds with fibrovascular cores and can also be in a papillary-trabecular or papillary-solid architectural pattern.	Aggressive	10–15%

Chromophobe	Patients >50 years Birt–Hogg–Dube syndrome	Hypovascular spoke-wheel contrast enhancement Indistinguishable from oncocytomas	Smaller eosinophilic and larger pale neoplastic cells with wrinkled nuclei and perinuclear halos arranged in a predominantly solid architectural pattern.	Favorable, low mortality	5–11%

Collecting duct	Male-to-female ratio: 2 : 1 Patients: fourth and fifth decades	Heterogeneous (necrosis, hemorrhage, and calcification) infiltrative growth Originates from the medullary center	High-grade neoplastic cells with variable architectural patterns ranging from cribriform, papillary, solid, and tubular. Prominent stromal desmoplasia.	Very aggressive, 1/3 metastatic on diagnosis	1%

Renal medullary	Associated with sickle cell trait Male-to-female ratio: 2 : 1 Mean age: 22 years	Hypovascular Heterogeneous (hemorrhage and necrosis) Liver and lung metastasis	High-grade eosinophilic, neoplastic cells in varying architectural patterns. Cribriform pattern is the most common. Prominent desmoplastic stroma.	Very aggressive Mean survival: 15 weeks	1%

Multiloculated cystic	Mean age: 50 years Predominate in women with male-to-female ratio 1 : 4	Largely cystic lesions, <25% solid	Multiple cysts with thin septa, lined by clear cells.	Indolent, favorable prognosis	Less than 1%

Tubulocystic	Patients >50 years Male-to-female ratio: 7 : 1	Predominately cystic lesion with thin septa	Purely tubular and cystic growth pattern. Cysts lined by a single layer of neoplastic cells with hobnail appearance.	Largely indolent, rare reports of metastatic disease	Rare (less than 100 cases reported)

## Abbreviations

TCC: Tubulocystic renal cell carcinoma
RCC: Renal cell carcinoma
ESRD: End-stage renal disease
CT: Computed tomography
CD: Cluster of differentiation
CK: Cytokeratin
EMA: Epithelial membrane antigen.
